# Retracted: Effect of Salinity on Biomass Yield and Physiological and Stem-Root Anatomical Characteristics of Purslane (*Portulaca oleracea* L.) Accessions

**DOI:** 10.1155/2019/9458681

**Published:** 2019-08-08

**Authors:** BioMed Research International

The article “Effect of Salinity on Biomass Yield and Physiological and Stem-Root Anatomical Characteristics of Purslane (*Portulaca oleracea* L.) Accessions” [1] has been retracted as it was found to contain duplicated figures and text similarity. Comments on PubPeer noted concerns with Figures 8, 9, and 11 [2]. In Figure 8, the 0dS m^−1^ panel is identical to the 8dS m^−1^ panel, but with a lower contrast. In Figure 9, the five panels are identical, but rotated or magnified.

In addition, we found that the 8dS m^−1^ panel in Figure 10 is identical to the 32dS m^−1^ panel in Figure 12. These concerns are highlighted in the Supplementary File (available here).

We also found a similar article published in* Biological Research* by the same group of authors [3]. The article's full details are as follows: Md. Amirul Alam, Abdul Shukor Juraimi, M. Y. Rafii, Azizah Abdul Hamid, Farzad Aslani and M. A. Hakim, “Salinity-induced changes in the morphology and major mineral nutrient composition of purslane (Portulaca oleracea L.) accessions,”* Biological Research*, 2016 49:24, https://doi.org/10.1186/s40659-016-0084-5. We sought the authors' clarification and they stated they have lost the data.

## Supplementary Materials

Supplementary Materials The file includes the problem figures that were spotted by Hindawi and in PubPeer Comments.
