# Supplementary material for: Retracted: Effect of Salinity on Biomass Yield and Physiological and Stem-Root Anatomical Characteristics of Purslane (Portulaca oleracea L.) Accessions
Source: Biomed Res Int. 2019 Aug 8;2019:9458681. doi: 10.1155/2019/9458681 (PMC6702845; doi:10.1155/2019/9458681)
Supplement: Supplementary Materials — The file includes the problem figures that were spotted by Hindawi and in PubPeer Comments. [file 9458681.f1.docx]

**105695. Problem Figures**

Figures 8 and 9


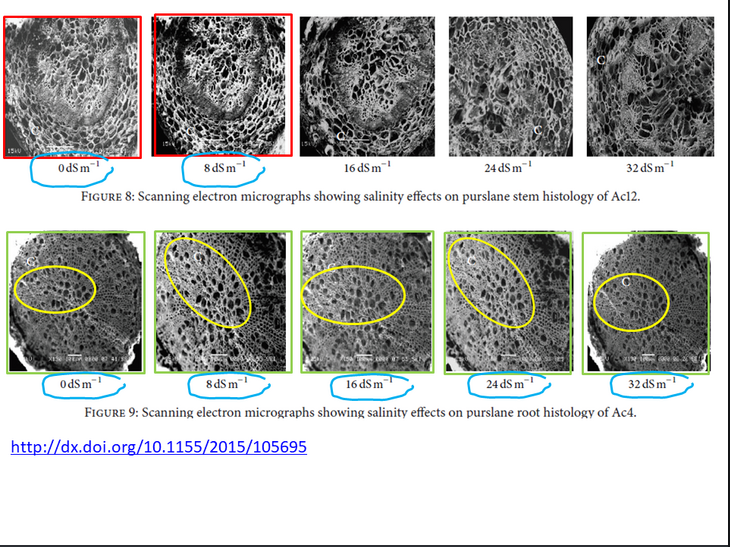


**Figure 8: Scanning electron micrographs showing salinity effects on purslane stem histology of Ac12.**

**Figure 9: Scanning electron micrographs showing salinity effects on purslane root histology of Ac4.**

Figure 11


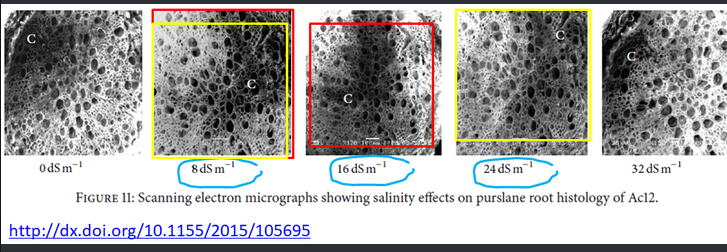


**Figure 11: Scanning electron micrographs showing salinity effects on purslane root histology of Ac12**

Figure 10 and 12


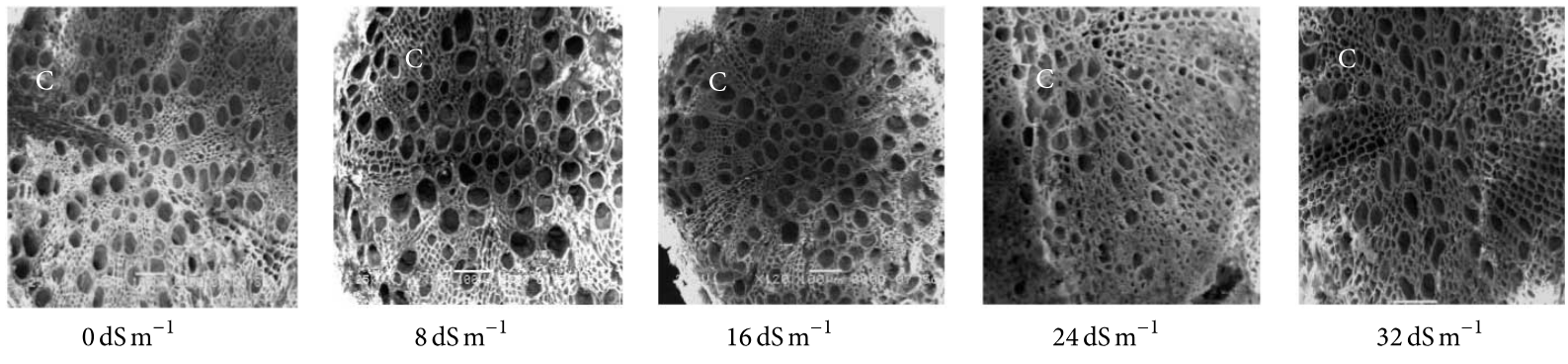


**Figure 10: Scanning electron micrographs showing salinity effects on purslane root histology of Ac10.**


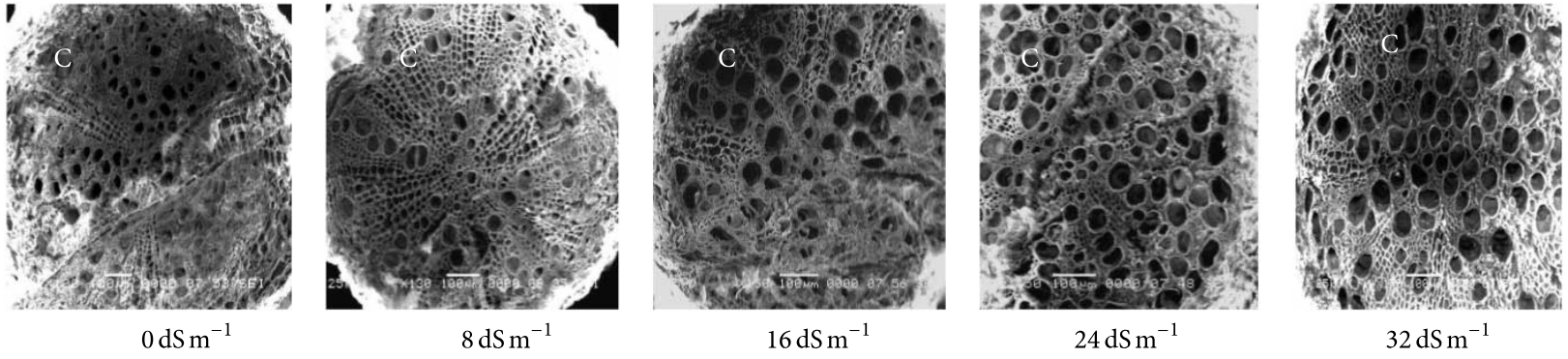


**Figure 12: Scanning electron micrographs showing salinity effects on purslane root histology of Ac13.**
